# Microbial inhibition of oral epithelial wound recovery: potential role for quorum sensing molecules?

**DOI:** 10.1186/s13568-015-0116-5

**Published:** 2015-05-21

**Authors:** Tine De Ryck, Eline Vanlancker, Charlotte Grootaert, Bart I. Roman, Laurens M De Coen, Isabel Vandenberghe, Christian V Stevens, Marc Bracke, Tom Van de Wiele, Barbara Vanhoecke

**Affiliations:** Laboratory of Experimental Cancer Research (LECR), Ghent University, 9000 Ghent, Belgium; Laboratory of Microbial Ecology and Technology (LabMET), Ghent University, Coupure Links 653 Building A, 9000 Ghent, Belgium; Laboratory of Food Chemistry and Human Nutrition, Ghent University, 9000 Ghent, Belgium; SynBioC, Department of Sustainable Organic Chemistry and Technology, Ghent University, 9000 Ghent, Belgium; L-Probe, Ghent University, 9000 Ghent, Belgium

**Keywords:** Epithelial wound healing, Co-culture model, *Klebsiella oxytoca*, Quorum sensing, Monocultures

## Abstract

Awareness of the impact of microbiota in both health and disease is growing. Using a new in vitro oral mucosa co-culture model, we recently showed a clear inhibition of epithelial wound healing in the presence of an oral microbial community. In this paper, we have used the same model in combination with specific oral microbial species to obtain a better insight into the role of the oral microbiota in wound healing. Monocultures of *Klebsiellaoxytoca* and *Lactobacillus salivarius* significantly inhibited wound healing with ~20%, whereas *Streptococcus mitis* and *S. oralis* enhanced the healing process with ~15% in 24 h. Yet, neither *S. oralis* or *S. mitis* were able to counteract the inhibitory effects from *K.oxytoca* on wound healing. Other tested microbial species had no effect on wound healing. Apart from this species-dependency, the inhibitory effect on wound healing depended on a microbial threshold concentration. Further mechanistic experiments with *K.oxytoca* excluded different microbial factors and hypothesized that quorum sensing molecules might play a role in the inter-kingdom signalling during wound healing. These results are important for the development of new strategies for the management of (infected) wounds and ulcerations.

**Electronic supplementary material:**

The online version of this article (doi:10.1186/s13568-015-0116-5) contains supplementary material, which is available to authorized users.

## Introduction

Microbiota are omnipresent in the human body, where they are colonizing the skin, mouth, nose, ears, vagina and the intestinal tract, each with their particular community (Bik 2009). Although awareness of the involvement of microbiota in human health and disease is growing, research in the area of non-infectious host–microbe interactions is complicated by the lack of suitable models due to the cytotoxicity of microbiota towards host cells in long-term experiments (Marzorati et al. 2011). To circumvent this issue, most studies report the results of experiments with limited co-culture times (<4 h) or using microbial supernatant instead of living cultures. In this study, we applied our recently developed co-culture model to study host–microbe interactions during wound healing in non-infectious conditions (De Ryck et al. 2014). By means of a semi-permeable membrane, microbiota and epithelial cells are physically separated, while metabolites and other secreted factors can pass the membrane. This enables the study of indirect crosstalks between the microbiota and the host epithelium for a timeframe up to 72 h.

Wound healing and re-epithelisation are a set of complex processes that are initiated upon tissue injury. In many studies, oral wound healing is considered as a representative model for scarless and fast healing (Enoch and Stephens 2009; Glim et al. 2013). Edwards and Harding (2004) reviewed the role of microbiota in chronic wounds, marking an important distinction between microbial colonization and infection. They showed that the presence of low numbers of microbiota in the injured tissue could improve the wound healing process, whereas lesions infected with large amounts of microbiota are characterised by poor healing. It is still largely unknown which microbial species, factors or activities have an impact on epithelial wound healing. Already in 1994, Okada showed that the healing of longitudinal skin incisions was enhanced in the presence of normal intestinal microbiota in comparison to wound healing in germ free mice (Okada 1994). In contrast, Laheij et al. (2013) found that wounds infected with *Porphyromonas gingivalis*, *Prevotella nigrescens* and secretions of *P. gingivalis* strongly inhibited cell migration, whereas weaker inhibitory effects were found for *Prevotella intermedia*, *Tannerella forsythia* and *Streptococcus mitis*. These results confirm our previous observation that the oral microbiota may be involved in delayed wound healing (De Ryck et al. 2014).

Factors that have been shown to have an impact on wound healing are microbial cell wall components such as lipopolysaccharides (LPS) (Koff et al. 2006), microbial metabolites such as short chain fatty acids (SCFAs) (Wilson and Gibson 1997) and other secreted factors such as the toxin C3-transferase from *Clostridium botulinum* (Aepfelbacher et al. 1997).

Previously, we showed an overall negative impact of oral microbiota derived from a buccal swab on wound healing of oral-derived epithelial cells (De Ryck et al. 2014). In this study, we aim to obtain a better understanding of the underlying mechanisms using mono- and mixed cultures of species known to be present in the oral cavity in healthy or diseased states.

## Materials and methods

### TR146 cell line

The TR146 cells, an oral squamous carcinoma cell line derived from a local lymph node metastasis, were kindly provided by Clare Hall Laboratories (Cancer Research UK). The cells were cultured in Dulbecco’s modified Eagle’s Medium (DMEM) (Gibco, Merelbeke, Belgium) supplemented with 10% heat-inactivated fetal bovine serum (Greiner bio-one, Belgium), 22.8 µg/mL penicillin–streptomycin (5,000 U/mL; Gibco, Merelbeke, Belgium) and 2.5 µg/mL amphotericin B (Bristol-MyersSquibb, Braine-l’Alleud, Belgium) at 37°C and 10% CO_2_. Cells were regularly checked for mycoplasma contamination (MycoAlert Mycoplasma Detection kit; Lonza, Rockland, USA).

### Microbial cultures

Different monocultures were obtained from the BCCM^™^/LMG bacteria collection (Ghent, Belgium). *Streptococcus salivarius* (LMG11489), *S. oralis* (LMG 14553), *S. mitis* (LMG 14557), *S.pyogenes* (LMG 15868) and *K. oxytoca* (LMG 3055) were cultured in BHI broth (Sigma-aldrich, Diegem, Belgium), *L. salivarius* (LMG 9477), *L. oralis* (LMG 9848) and *L. plantarum* (LMG 9211) were cultured in MRS broth (Sigma-aldrich, Diegem, Belgium) and *Neisseria mucosa* (LMG 5136) was cultured in heart infusion broth (Sigma-aldrich, Diegem, Belgium).

*Klebsiella oxytoca* AHC-6 (WT), *K. oxytoca* AHC-6 Mut89 (ΔnspB mutant) and *K. oxytoca* AHC-6 Mut89 + nspB (pACYC184; complementation) were kindly provided by Prof. E. Zechner (Institute of Molecular Biosciences, University of Graz, Graz, Austria) (Schneditz et al. 2014). All strains were cultured in CASO broth (Sigma-aldrich, Diegem, Belgium) supplemented with 50 µg/mL kanamycin or 30 µg/mL chloramphenicol for the mutant and complementation strain, respectively.

For microbial enumeration, suspensions were plated using the microdilution plating method, on BHI agar plates, MRS agar plates or CASO agar plates (BHI/MRS/CASO broth: Sigma-aldrich, Diegem, Belgium; 15% agar: BD, Erembodegem, Belgium) and incubated at 37°C. When plating mixed cultures, selective BHI agar plates with Listeria mono Selective Supplement I (Sigma-aldrich, Diegem, Belgium) were used for selectively culturing *Streptococci* at 37°C, whilst *K. oxytoca* was enumerated after incubating standard BHI plates at room temperature, as *Streptococci* were not able to grow at room temperature.

### Chemicals

A filter-sterilized stock solution of 5 mg/mL sodium d-lactate (Sigma-aldrich, Diegem, Belgium) was prepared in serum-free, antibiotics-free DMEM, which was further diluted in serum-free, antibiotics-free DMEM during the experiments to obtain the desired concentrations (100, 500, 1,000, 5,000 µg/mL).

The quorum sensing molecule *N*-(3-oxododecanoyl)-l-homoserine lactone (Sigma-aldrich, Diegem, Belgium) was dissolved in DMSO to obtain stock solutions of 15, 30, 45 and 60 mM. For experiments, further dilutions (1:500) in DMEM without serum and antibiotics were prepared.

For the synthesis of the pyrrolobenzodiazepine tilivalline, we followed the protocol described by Schneditz et al. (2014). However, we used the freeze–pump–thaw method (3×) instead of the vacuum/N_2_ cycle (3×) for degassing the reaction. In this way a yield of 57% was obtained compared to the previously reported 38% (Schneditz et al. 2014).

For the in vitro experiments, stock solutions of 100, 50, 10, 5 and 1 mM tilivalline were prepared in DMSO, which were further diluted (1:1,000) in DMEM without serum and antibiotics.

### Co-culture model

To co-culture microbiota and TR146 epithelial cells in non-infectious conditions, we used our recently published model (De Ryck et al. 2014). Briefly, 75 µL of an agar/mucin solution [5% porcine mucin type III (Sigma-aldrich, Diegem, Belgium), 0.8% agar (BD, Erembodegem, Belgium)] was brought on the porous membrane (0.4 µm) of a 24-well plate Transwell^®^ system (Corning Inc., NY, USA) and allowed to solidify for at least 30 min after which 20 µL of a microbial suspension was spotted on top of this agar/mucin layer (apical compartment). In the basal compartment, a monolayer of epithelial cells was grown and a wound scratch assay was performed as described below. During co-culture, the inserts with the microbiota were transferred into the wells with the wounded epithelial cells. For experiments with pre-incubation, inserts with microbiota were incubated for 4 h in wells filled with serum-free, antibiotics-free DMEM without epithelial cells, prior to their transfer into the wells with wounded epithelial cells.

### Wound scratch assay

For the wound scratch assays, TR146 epithelial cells were seeded (300,000 cells/well) in a 24-well plate (in absence of microbiota; Nunc Thermo Scientific, Erembodegem, Belgium) or in a 24-well plate Transwell^®^ system (co-culture model; Corning Inc., NY, USA) after labelling with DiI cell labelling solution (Life technologies Europe, Ghent, Belgium). At the start of the experiment a scratch was created with a sterile plastic pipette tip, all medium was discarded to remove cellular debris and fresh serum-free, antibiotics-free DMEM was added on the cells. Micrographs of selected points along the wounds were taken with an automated fluorescence microscope (Zeiss Axiovert 200M) at the start of the experiment and after 24 h of incubation at 37°C and 5% CO_2_.

In experiments using conditioned medium, the filter-sterile basal co-culture medium of experiments with *K. oxytoca* [inoculum concentration: 6 log colony forming units (CFU)/mL] was used and added on top of wounded epithelial layers. At the end of each experiment, cell viability was checked by use of an MTT assay, which was performed as described previously (De Ryck et al. 2014).

### Cytokine analysis

For cytokine analysis, we applied the Luminex platform (R&D Systems Europe, Abingdon, UK). By use of the multiplex kits (R&D Systems Europe, Abingdon, UK), concentrations of IL-1β, IL-6, TNF-α and Rantes in the basal conditioned medium of the co-culture model were determined following the manufacturer’s protocol.

### Lactate analysis

For the determination of l- and d-lactate concentrations in the co-culture media, we used the d-lactic acid/l-lactic acid (UV method) kit of R-biofarm (Roche, Vilvoorde, Belgium) as described previously (De Ryck et al. 2014).

### Glucose analysis

By use of the CMA 600M Microdialysis Analyser (CMA Microdialysis AB, Solna, Sweden), glucose concentrations present in the basal conditioned medium were determined following the manufacturer’s protocol using the glucose reagent (CMA Microdialysis AB, Solna, Sweden; Barham and Trinder 1972).

### Effects of glucose on wound healing

To test the effect of glucose on wound healing, different mixtures of serum-free, antibiotics-free DMEM with low glucose (1 g/L glucose; Gibco, Merelbeke, Belgium) and high glucose (4.5 g/L glucose; Gibco, Merelbeke, Belgium) were used.

### Fractionated conditioned medium

Basal medium of previous experiments in presence or absence of *K. oxytoca* was collected and sequentially fractionated using Amicon centrifugal filters (pore size 3 and 10 kDa; Merck Millipore, Overijse, Belgium) (Additional file 1: Figure S1a). The wound healing capacity in presence of the control-conditioned medium (absence of *K.oxytoca*) was compared to the control-conditioned medium in which particular fractions (x > 10 kDa; 10 kDa > x > 3 kDa) were interchanged with the same fraction of *K.oxytoca*-conditioned medium. For the fraction below 3 kDa, the control-conditioned fraction <3 kDa was compared to the *K.oxytoca*-conditioned fraction <3 kDa (Additional file 1: Figure S1b).

### Proteinase K treatment of conditioned medium

Conditioned medium of *K. oxytoca*-exposed cells was treated with proteinase K (200 µg/mL; Sigma-aldrich, Diegem, Belgium) during 1 h at 37°C. After cooling down on ice for 5 min, the medium was boiled at 98°C for 10 min and centrifuged during 10 min at maximum speed to inactivate the enzyme. The supernatant was further filter-sterilised before use in a wound scratch assay. Proteinase activity and subsequent inactivation were checked using bovine serum albumin (Sigma-aldrich, Diegem, Belgium) dissolved in DMEM as a control protein.

### Matrix-assisted laser desorption/ionization time-of flight (MALDI-TOF) analysis

For MALDI-TOF analysis of the conditioned medium fraction <3 kDa, samples of control-conditions and *K. oxytoca*-exposed cells (<3 kDa) were treated with SPE STRATA X (Phenomex, Utrecht, The Netherlands). Different fractions (drain, washing steps of 5, 10 and 50% methanol and the eluate with 50/50 ACN/MeOH) were concentrated using a vacuum centrifuge (Speedvac SC110 concentrator, NY, USA). MS and tandem MS spectra were acquired on a 4800 Plus MALDI TOF/TOF analyzer (ABSCIEX, Framingham, USA), using the delayed extraction and reflector technologies in the positive ion mode. Instrument calibration was performed using the 4700 mass standard kit from Applied Biosystems (Life Technologies, Ghent, Belgium).

### Tris–Tricine gel analysis

The medium fraction <3 kDa of control conditions and *K. oxytoca*-exposed cells was concentrated (10×) by lyophilisation followed by dissolving in loading buffer [200 mM Tris–HCl (pH 6.8), 2% SDS, 40% glycerol, 0.04% Coomassie brilliant blue, 2% β-mercapto-ethanol]. Peptides were separated on a Mini protean^®^ Tris–Tricine precast gel (Bio-Rad laboratories, Eke, Belgium) as described in the manufacturers protocol. After fixation in 50% methanol and 10% acetic acid during 30 min, the gel was stained for 1 h in a Coomassie Brilliant blue R-250 staining solution (Bio-Rad laboratories, Eke, Belgium). Destaining of the gel was performed in a 5% methanol/7% acetic acid/H_2_O solution until the desired background was obtained.

### Statistics

For statistical analysis of the different experiments, Shapiro–Wilk analysis was used to check normality of the data. Depending of the normality and the experiment Student’s t tests, ANOVA, Kruskal–Wallis or Mann–Whitney U analyses were performed. Bonferroni correction was used when performing multiple comparisons. All analyses were performed using the SPSS Statistics 22 software package and differences were considered significant at the *p* < 0.05 level.

## Results

### Microbial effects on wound healing: mono- and mixed cultures

**Figure 1:**
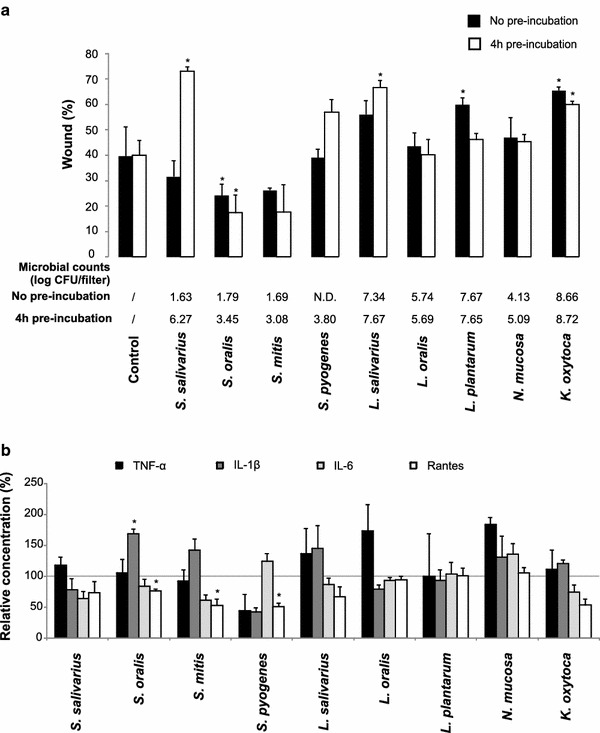
**a** Wound healing capacity of TR146 epithelial cells confronted with inserts containing 5.5–6 log CFU of monocultures of different oral species. The relative area of the wound after 24 h is plotted (mean + SD; *p < 0.05). The *black bars* represent data from experiments performed without pre-incubation of the microbial cells on the insert, the *white bars* represent data from experiments with a 4 h pre-incubation step of the microbiota before confrontation with the epithelial cells. Mean microbial counts of the different species present on the filters after 24 h of co-culture with TR146 epithelial cells are depicted (*N.D.* = not detected). **b** Cytokine analysis of the basal conditioned medium. The microbial monocultures of different oral species were pre-incubated for 4 h before confrontation with the TR146 cells for 24 h. Relative concentrations are shown (mean + SD; *p < 0.05).

Wounded TR146 epithelial cells were exposed to 5.5–6 log CFU of *S. salivarius*, *S. oralis*, *S. mitis*, *S. pyogenes*, *L. salivarius*, *L.oralis*, *L. plantarum*, *N.mucosa* or *K. oxytoca* monocultures for 24 h (no pre-incubation). These microbiota are all known to be present in the oral cavity, with Streptococci as most important habitant. *S. salivarius*, *S.oralis* and *S. mitis* are frequently isolated from the healthy oral cavity. *S. pyogenes* was added to the test panel as a known virulent *Streptococcus* spp. causing for example pharyngitis. *Lactobacillus* spp. are less abundant in the oral cavity (<1%), but were chosen for their ability to produce lactic acid. Furthermore, *N.mucosa* is a microbe known to colonize the mucosal surfaces within the oral cavity and *K. oxytoca* is mainly isolated from the oral cavity after irradiation for head and neck cancer (Marsh and Martin 1999). Remarkably, wound healing was only significantly reduced in the presence of *L.plantarum* (−20%; p = 0.003) and *K.oxytoca* (−26%; p = 0.007). In contrast, *S. oralis* significantly enhanced wound closure after 24 h (+15%; p = 0.022) and *S. mitis* showed a similar trend (+13%; p = 0.054) (Figure 1a—black bars; Additional file 1: Figure S2). All p values are listed in Additional file 1: Table S1a.

The different outcome depending on the type of species was not the result of reduced epithelial viability (Additional file 1: Figure S3), but could be explained by the fact that some species grew better on the agar/mucin substrate during co-culture than others. Enumeration of the microbiota after 24 h of co-culture with TR146 cells indeed revealed important differences in survival between the test species. The number of *Streptococci* was strongly reduced to ~1 log CFU during co-culture, whereas lactobacilli and *K. oxytoca* grew steadily with another 2–3 log units (Figure 1a; Additional file 1: Table S1b).

In order to evaluate whether immediate confrontation of the microbiota with TR146 cells was detrimental for the establishment of a biofilm on an agar/mucin substrate, monocultures of test species were added to the substrate 4 h prior to confrontation with the wounded TR146 monolayers (4 h pre-incubation). When we included this pre-incubation step, also *S. salivarius* (−33%) and *L. salivarius* (−27%) had a significant negative impact on wound healing (p < 0.001). In contrast, *S. oralis* consistently and significantly stimulated wound closure after 24 h (+22.5%; p = 0.029), while *S. mitis* displayed a trend to enhance wound healing (+22%; p = 0.081; Additional file 1: Table S1a) (Figure 1a; white bars). All *Streptococci* were at least present at 3 log CFU/filter after 4 h of pre-incubation (Figure 1a; Additional file 1: Table S1b). From these experiments, we can conclude that (1) the effect of wound recovery is species-dependent and (2) there seems to be a strong correlation between the number of *S. salivarius* and the effect on wound healing.

Cytokine analysis of the basal medium collected after 24 h of co-culture revealed no direct link between the type and concentration of cytokines released and the effects on wound healing. While a significant increase of IL-1β was observed for *S. oralis* (p = 0.008), no significant changes as compared to levels in the control conditions could be noticed for the other test species. Rantes was significantly reduced in presence of *S.oralis, S. mitis* and *S. pyogenes* (p = 0.017; p = 0.020; p = 0.016, resp.) but not in presence of the other species (Figure 1b; Additional file 1: Table S2). TNF-α and IL-6 levels remained largely unchanged. Overall, the microbiota-induced release of cytokines in TR146 is low in our model, which is probably due to the absence of real infectious conditions.

**Figure 2:**
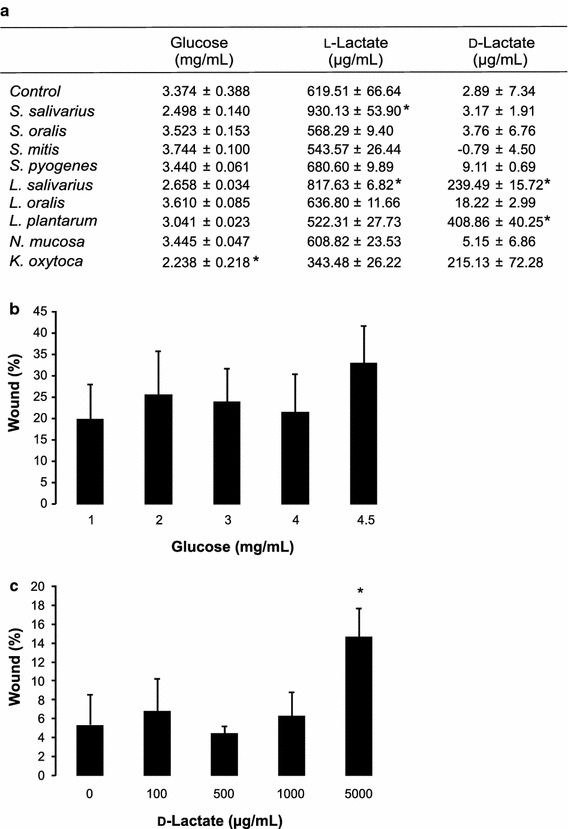
**a** Concentrations of glucose, l- and d-lactate found in the basal cell culture medium after 24 h of co-culture of the TR146 cells with different oral microbial species (mean ± SD; *p < 0.05). Microbiota were pre-incubated for 4 h on the insert, before they were exposed to the wounded TR146 cells. **b** Wound healing capacity of TR146 cells in cell culture medium with different glucose concentrations (mean + SD; *p < 0.05). **c** Wound healing capacity of TR146 cells treated with different d-lactic acid concentrations (mean + SD; *p < 0.05).

As microbial growth requires energy, microbial metabolism will reduce the glucose concentrations present in the co-culture medium (with pre-incubation). The glucose concentration in the co-culture medium of *K. oxytoca*-exposed cells was significantly decreased to 2.238 mg/mL (p = 0.009) (Figure 2a), whereas a non-significant drop was noticed with *S. salivarius* and *L. salivarius* (p = 0.081). However, further experiments using glucose concentrations ranging from 1 to 4.5 g/L (standard cell culture medium), showed no differences in wound healing (p = 0.148; Figure 2b). This made us conclude that glucose-depletion by microbial activity is probably not the underlying mechanism for wound healing inhibition.

We further questioned if the release of acids like l- and d-lactic acid in the co-culture medium (with pre-incubation) could explain the inhibiting effect of certain species on wound healing (Figure 2a). At least for *S.salivarius* and *L. salivarius* higher levels of l-lactate (930.1 µg/mL, p = 0.027; 817.6 µg/mL, p = 0.027 resp.) were found compared to the control condition (without microbiota; 619.5 µg/mL). However, no elevated levels were observed for *K. oxytoca*. d-lactic acid concentrations were dramatically increased in presence of *L. salivarius* and *L. plantarum* (239.5 µg/mL, p = 0.027; 408.9 µg/mL, p = 0.027 resp.) and, although not significantly, also in presence of *K. oxytoca* (215.1 µg/mL, p = 0.153). Exogenous d-lactic acid only showed inhibiting effects on wound healing at concentrations starting from 5,000 µg/mL (Figure 2c). Previously, lactic and acetic acid were found to inhibit wound healing starting at 1,800 µg/mL and thus not at concentrations that were found in the model (De Ryck et al. 2014). Together, these results indicate that acids do not play a major role in the effect on wound healing.

**Figure 3:**
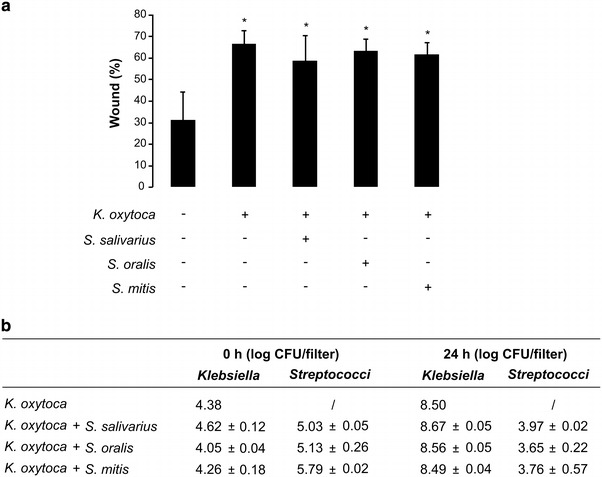
**a** Wound healing capacity of TR146 epithelial cells confronted with *K. oxytoca* alone or mixed with *S. salivarius*, *S. oralis* *or S. mitis* (mean + SD; *p < 0.05). **b** Microbial counts of *K. oxytoca* or mixtures of *K. oxytoca* with *S.* *salivarius*, *S. oralis* or *S. mitis* present on the insert at time 0 and after 24 h of co-culture with TR146 epithelial cells (mean ± SD).

In addition to monocultures, we also evaluated the effect of mixed cultures of *S.salivarius*, *S.oralis* or *S. mitis* with *K. oxytoca* (no pre-incubation) on the wound healing of TR146 cells. Overall, we found a dominant negative effect on wound healing, probably due to the presence of *K. oxytoca*. Whereas monocultures of *S. oralis* and *S. mitis* had a stimulating effect on wound healing, these two strains were completely unable to counteract the inhibitory effect of *K. oxytoca* towards wound healing (Figure 3a). This may be explained by the 5 log higher microbial counts for *Klebsiella* compared to *Streptococci* after 24 h (Figure 3b).

### The involvement of small microbial molecules in wound healing

Due to its dominating negative effect and its well-known virulence, we chose *K. oxytoca* as a model organism to further elucidate the mechanism underlying its effects on wound healing.

**Figure 4:**
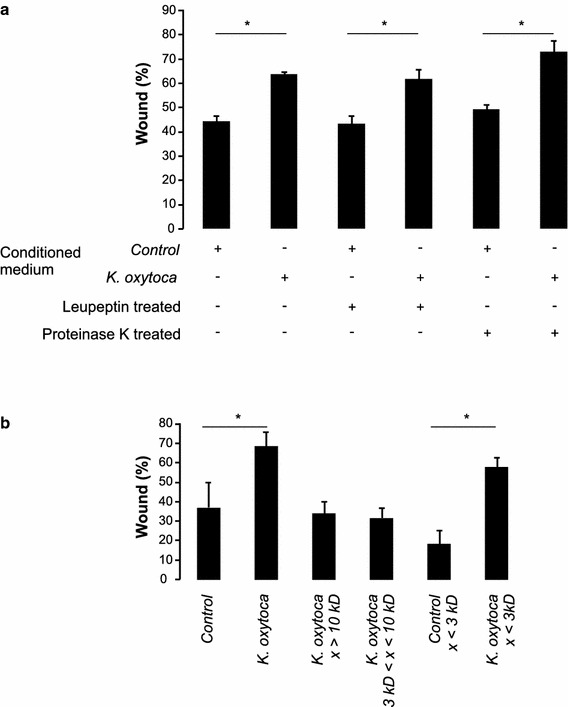
**a** Wound healing capacity of TR146 epithelial cells in presence of the conditioned medium of *K. oxytoca*-exposed TR146 cells (mean + SD; *p < 0.05). Leupeptin- or proteinase K-treatment of the conditioned medium was performed as described in “Materials and methods”. **b** Wound healing capacity of TR146 epithelial cells in presence of different fractions of the *K. oxytoca*-conditioned medium (mean + SD; *p < 0.05). Full conditioned medium was tested together with three different fractions >10 kDa, 3 kDa < x < 10 kDa and <3 kDa.

As shown in Figure 4a, the basal conditioned medium collected after 24 h of co-culture of TR146 cells and *K. oxytoca*, was also able to exert inhibitory effects on wound healing. This suggests an inhibitory effect of a cellular or microbial factor present in the co-culture medium. Treatment of the conditioned medium with leupeptin (a protease inhibitor) or proteinase K (a broad-spectrum serine protease) did not significantly alter this inhibitory effect (p_control-*K. oxytoca*_ < 0.001 in all three cases) indicating that the effect is probably not due to the activity of a protease or a protein (Figure 4a). Next, we tested three different fractions of the *K. oxytoca*-conditioned medium. Whilst the protein fraction >10 kDa and the fraction between 3 and 10 kDa of the *K. oxytoca*-conditioned medium showed no inhibitory effect (p = 1), fully *K. oxytoca*-conditioned medium (unfractionated) and the fraction <3 kDa caused a significant reduction in wound healing (p < 0.001) (Figure 4b; Additional file 1: Figure S1). We therefore concluded that a small molecule or peptide present in the fraction <3 kDa of the *K. oxytoca*-conditioned medium is likely to be responsible for the effect on wound healing. Unfortunately, separation on a Tris–glycine gel and visualisation by Coomassie staining of the <3 kDa fraction of both the control- and *K. oxytoca*-conditioned medium did not reveal clear differences (data not shown). Using MALDI-TOF analysis, we could identify one peptide that was less abundant in the control sample compared to the eluate of the *K.oxytoca*-conditioned sample (* in Additional file 1: Table S3). Yet, further MS/MS analysis of this peptide pointed to background artefacts or medium compounds.

**Figure 5:**
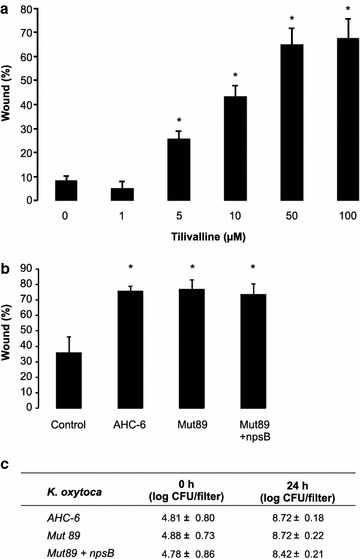
**a** Wound healing capacity of TR146 cells treated with different concentrations of tilivalline (mean + SD; *p < 0.05). **b** Wound healing capacity of TR146 cells exposed to *K. oxytoca* AHC-6 wild type, the ΔnpsB mutant Mut89 unable to produce tilivalline and the Mut89 with npsB complementation (mean + SD; *p < 0.05). **c** Microbial counts of the different *K. oxytoca* strains (AHC-6, Mut 89, Mut89 + npsB) present on the insert at time 0 and after 24 h of co-culture with TR146 epithelial cells (mean ± SD).

Altogether, these results indicate that at least for *K. oxytoca* the inhibitory factor is likely to be a small molecule different from an acid or a small peptide. One such molecule produced by *K. oxytoca* and recently reported to be toxic for epithelial cells is tilivalline (Schneditz et al. 2014). Therefore, we studied the epithelial wound healing capacity in the presence of synthetic tilivalline and found a significant inhibition when tilivalline was present at concentrations starting from 5 µM (p_0–1 µM_ = 0.325, p_0–5 µM_ = 0.01, p_0–10 µM_ = 0.01, p_0–50 µM_ = 0.01, p_0–100 µM_ = 0.01; Figure 5a). At these concentrations a higher amount of death, floating cells was observed, although the viability of the adherent cells was not affected (data not shown). To further investigate the potential of tilivalline, we performed co-culture experiments with different tilivalline-mutant *K. oxytoca* strains. Unlike the AHC-6 wild type and Mut89 + nspB strain, the Mut89 strain is unable to produce tilivalline (Schneditz et al. 2014). Unfortunately, all three tested *K. oxytoca* strains (AHC-6, Mut89 and Mut89 + npsB) inhibited epithelial wound healing significantly (p < 0.001), showing no differences between the three strains (p = 1) (Figure 5b, c). These results exclude the involvement of tilivalline in the microbial effects on epithelial wound healing.

**Figure 6:**
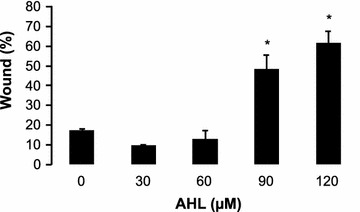
Wound healing capacity of TR146 cells treated with different concentrations of *N*-(3-oxododecanoyl)-l-homoserine lactone (AHL) (mean + SD; *p < 0.05).

Last, we tested the hypothesis that quorum sensing molecules might be involved in the inhibition of wound healing. We chose to test *N*-(3-oxododecanoyl)-l-homoserine lactone as this molecule has been shown to induce apoptosis in epithelial breast cancer cells (Li et al. 2004). Starting from 90 µM, a significant decrease in wound healing capacity was observed (p_90 µM_ = 0.012; p_120 µM_ = 0.02; Figure 6).

## Discussion

The human epithelium is the most important barrier for all physical, chemical or biological attacks on the human body. In case of acute damage, wound healing processes are very important to protect the inner tissues. Pathogenic infections of the wound are known to delay wound healing (Edwards and Harding 2004). Here we show that indirect host–microbe interactions are also able to modulate the epithelial wound healing process. Indirect exposure of epithelial cells to *K. oxytoca* has a significant negative effect on the healing process whereas no influence or an improvement of the healing was found after co-culture with e.g. *N.mucosa* and *S. oralis*, respectively. Inhibition of cellular migration without the direct exposure of epithelial cells to microbiota has previously been shown by the use of *Pseudomonas aeruginosa* or Peptostreptococcal supernatants (de Bentzmann et al. 2000; Stephens et al. 2003). In contrast, supernatant of *Escherichia coli* (strain DH5α) or *Citrobacter diversus* did not reveal pronounced effects on epithelial wound healing (de Bentzmann et al. 2000; Stephens et al. 2003), confirming our findings of a species-specific effect.

The microbial density in the wound seems to be a very important parameter. A low grade inflammatory response as a consequence of low concentrations of microbiota have been shown to accelerate wound healing (Häkkinen et al. 2000). Low numbers (10^2^) of *Staphylococcus aureus*, for example, have been shown to enhance local blood flow, while its enterotoxin A induces the accumulation of collagen hydroxyproline, both contributing to accelerated healing (Laato et al. 1990). Also microbial factors like phospholipase C and LPS have been found to modulate cell migration (Firth et al. 1997; Häkkinen et al. 2000; Koff et al. 2006). At 10 µg/mL, LPS stimulates wound healing, whereas higher concentrations appeared to be toxic (Koff et al. 2006), which was also confirmed in our model (data not shown). However, all these compounds have a size >3 kDa and could be excluded as modulating factors from *K.oxytoca* in this study. The importance of the number of microbiota required to induce inhibitory effects on wound healing also became clear in our study. Without pre-incubation, *S.salivarius* was unable to grow sufficiently on the agar/mucin substrate in order to obtain a negative effect on wound healing. However, after a pre-incubation period of 4 h, enough viable microbiota were present to exert an inhibitory effect on wound healing.

Since microbial growth appeared important in order to observe effects on wound healing, a time-lapse experiment with *K.oxytoca* was performed monitoring both microbial growth and epithelial wound healing after 8, 12 and 16 h. While microbial cultures in the apical compartment were fully grown after 8 h, the effect on wound healing was only seen after more than 12 h of incubation (data not shown). These results indicate that accumulation of (a) microbial metabolite(s) or molecule(s) is needed before wound healing is affected. SCFA and lactic acid are the most important microbial metabolites in carbon-rich environments. Although SCFA have been described to affect cellular migration of colon epithelial cells (Wilson and Gibson 1997), we previously showed that reduced healing was only found at concentrations higher than those detected in our model (De Ryck et al. 2014). Another metabolite that can negatively impact health when accumulated is lactic acid. The combination of high levels of d-lactic acid and low pH has been shown to increase epithelial inflammation (Hanstock et al. 2010). Although inflammatory reactions have been demonstrated to impair wound recovery in vivo, d-lactic acid supplementation in our model did not reduce wound healing up to tenfold higher concentrations than those found in the co-culture model. Finally, reduced concentrations of glucose, the most important source of carbohydrates in our model, did not explain the effects on wound healing. McDermott et al. (1998) have previously shown reduced migration when glucose concentrations in the media were raised. No reports of reduced healing due to glucose-shortage were found, thus eliminating the predominant role of glucose in reduced wound healing.

Specific proteins have been reported to affect epithelial migration or wound healing. Elastase for example, a serine proteinase produced by *Ps. aeruginosa*, has previously been reported as the causing agent for the reduction of airway epithelial wound repair (de Bentzmann et al. 2000). However, in our study, treatment with the serine proteinase inhibitor leupeptin did not restore the inhibitory effect of *K. oxytoca* on wound healing. Further, experiments with proteinase K showed no evidence for the involvement of a particular protein or peptide in the underlying mechanism. Our observations are supported by Halper et al. (2003), who also excluded proteins or peptides present in the supernatant of *Lactobacillus* as causing factors for the effect on wound healing.

Although it was already described in 1982 that *K. oxytoca* produces the pyrrolobenzodiazepine cytotoxin tilivalline (Mohr and Budzikiewicz 1982), only recently a direct link between tilivalline and antibiotic-associated haemorrhagic colitis has been described (Schneditz et al. 2014). In our study, tilivalline significantly inhibited the wound healing capacity of TR146 epithelial cells. However, no differences in the effects on wound healing were observed between the *K. oxytoca* tilivalline mutant strain, the wild type and the complementation strain. This suggests that its concentration present in the co-culture medium is probably too low to exert any effect and thus excludes this molecule from being responsible for *K. oxytoca*’s inhibitory effect on wound healing in our model.

Many microbiota use quorum sensing molecules to coordinate processes that are microbial concentration-dependent such as biofilm formation and virulence. Common classes of quorum sensing molecules are oligopeptides produced by Gram-positive bacteria and *N*-acyl-homoserine lactones (AHL) produced by Gram-negative bacteria (Miller and Bassler 2001). Recently, researchers proposed a role for quorum sensing molecules in the communication between microbiota and their host (Hughes and Sperandio 2008; Pacheco and Sperandio 2009). Due to the clear similarities between the bacterial quorum-sensing mechanisms and the metastatic process initiated by tumour cells, the use of quorum-sensing signalling peptides in oncology is now being investigated (Wynendaele et al. 2012). For example, *N*-3-oxo-dodecanoyl-homoserine lactone, a quorum sensing molecule secreted by *Ps.aeruginosa*, induces apoptosis in several breast cancer cell lines and enhances the production of interleukins (IL-6, IL-8) in bronchial epithelial cells (Li et al. 2004; Mayer et al. 2011; Smith et al. 2001). Our data indicate that this molecule also inhibits the wound healing capacity of the TR146 cells at physiologically relevant concentrations. Although the most common quorum sensing molecules found in the oral cavity are the oligopeptides and auto-inducer-2 molecules produced by Gram positive bacteria, Yin et al. (2012) recently isolated *N*-octanoyl-homoserine lactone and *N*-3-dodecanoyl-l-homoserine lactone, produced by *K.pneumoniae* residing on the dorsal surface of the tongue. Also, *K.oxytoca* isolated from patient blood samples was found to produce high amounts of AHL when grown in stationary conditions (Wang et al. 2006). Taken together, these data hypothesize that quorum-sensing molecules produced by *Klebsiella* may contribute significantly to the inhibitory effect on epithelial wound healing. Further research should elaborate on the direct effects of different quorum sensing molecules on epithelial wound healing.

In conclusion, we show a species-, and concentration-dependent effect on epithelial wound healing in our in vitro oral mucosa model. In our search for molecules involved in the crosstalk between microbiota and host cells during wound healing, we were unable to fully unravel the mechanism underlying the inhibitory effect of *K. oxytoca*. However, our preliminary results suggest that quorum sensing molecules might play a role in this process. This opens new thoughts in the field of the host–microbe interactions, which need to be addressed in the future.

## Additional files

Additional file 1:
**Supplementary figures and tables**
