# Supplementary material for: Microbial inhibition of oral epithelial wound recovery: potential role for quorum sensing molecules?
Source: AMB Express. 2015 May 21;5:27. doi: 10.1186/s13568-015-0116-5 (PMC4437994; doi:10.1186/s13568-015-0116-5)
Supplement: Additional file 1: — Supplementary figures and tables [file 13568_2015_116_MOESM1_ESM.pdf]

AMB express

Microbial inhibition of oral epithelial wound recovery: potential role for quorum sensing molecules?

Tine De Ryck[1], Eline Vanlancker[1,2], Charlotte Grootaert[3], Bart I. Roman[4], Laurens M. De Coen[4], Isabel Vandenberghe[5], Christian V. Stevens[4], Marc Bracke[1], Tom Van de Wiele[2*], Barbara Vanhoecke[2]

[1] Laboratory of Experimental Cancer Research (LECR), Ghent University, 9000 Ghent, Belgium.

[2] Laboratory of Microbial Ecology and Technology (LabMET), Ghent University, 9000 Ghent, Belgium.

[3] Laboratory of Food Chemistry and Human Nutrition, Ghent University, 9000 Ghent, Belgium

[4] SynBioC, Department of Sustainable Organic Chemistry and Technology, Ghent University, 9000 Ghent, Belgium

[5] L-Probe, Ghent University, 9000 Ghent, Belgium

*: Corresponding author:

Tom Van de Wiele, Laboratory of Microbial Ecology and Technology (LabMET), Coupure Links 653 Building A, 9000 Ghent, Belgium. Phone: +32 9 264 59 12, Fax: +32 9 264 62 48; Email: tom.vandewiele@ugent.be

Supplementary figures

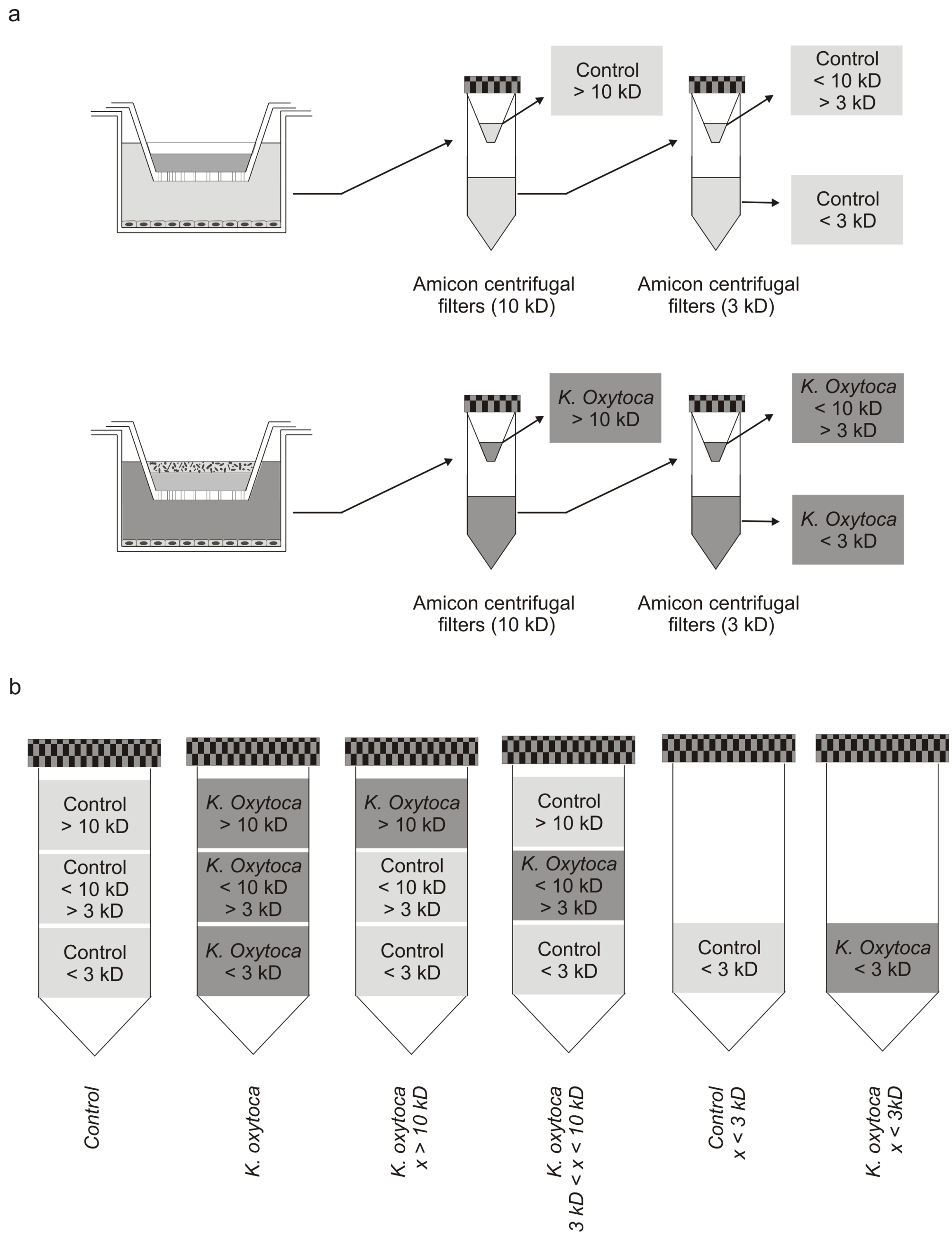

Figure S1a: Schematic overview of the fractionation of the control- and *K. oxytoca*-conditioned medium by use of Amicon centrifugal filters (3kD, 10kD; Merck Millipore, Overijse, Belgium). **b:** Replacement strategy of particular fractions in the control conditioned medium (light grey) with the same fractions of the conditioned medium of *K. oxytoca*-exposed cells (dark grey), in order to analyse the effect of different medium fractions on wound recovery.

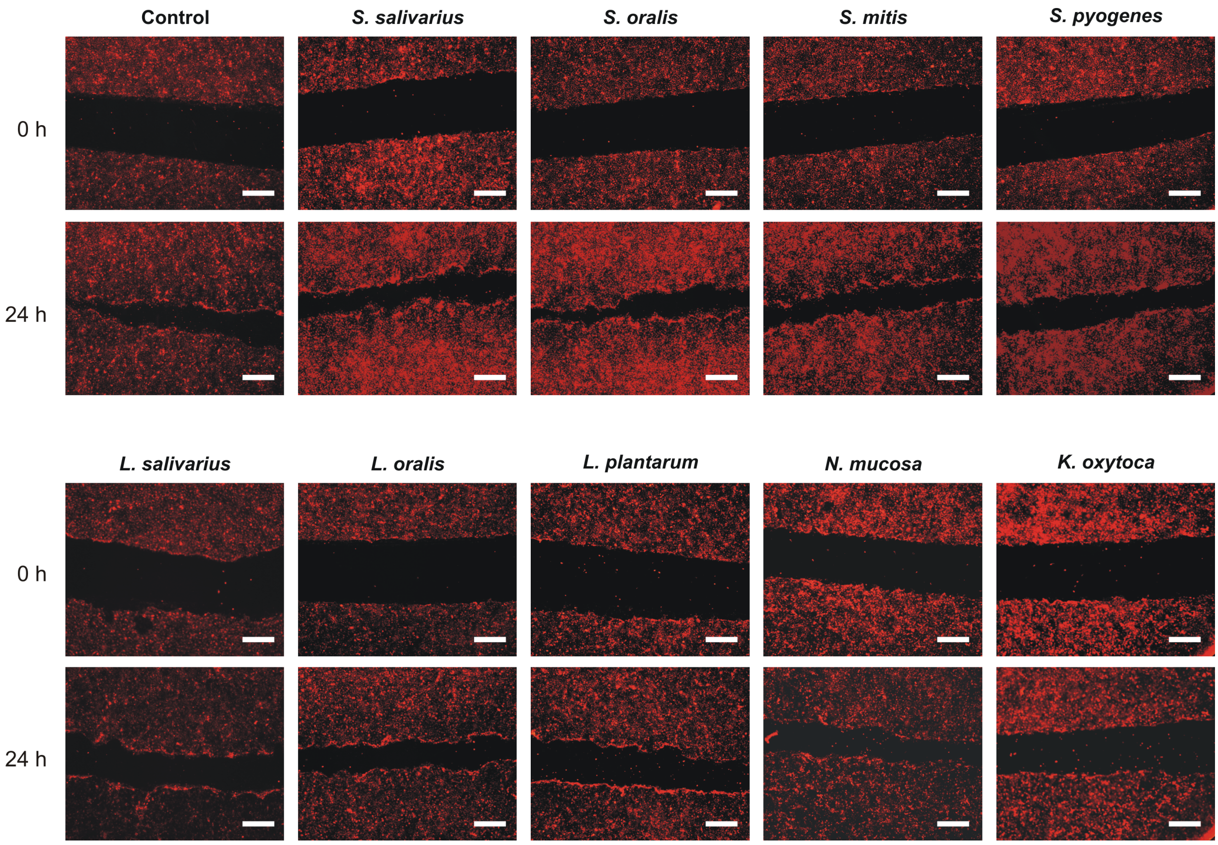

Figure S2: Representative micrographs of the wound healing capacity of TR146 cells at 0 h and after 24 h of co-incubation with monocultures of different oral species without pre-incubation (scale bar = 500 µm).

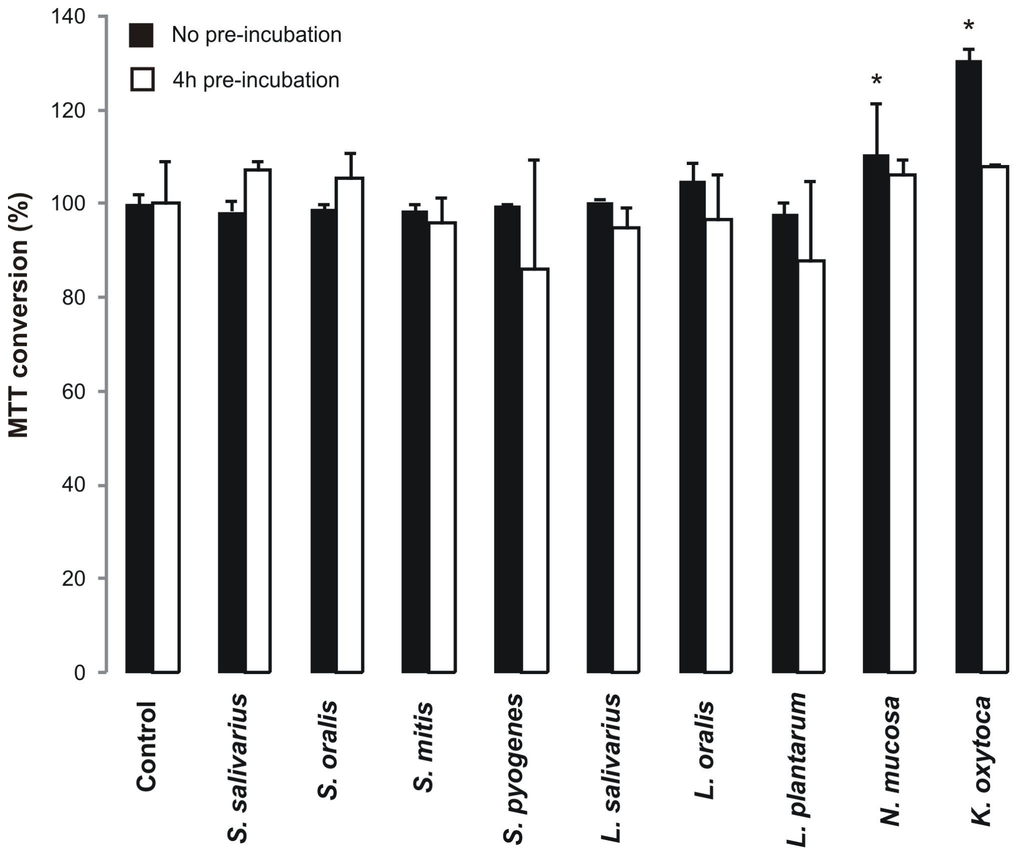

Figure S3: MTT conversion of TR146 cells after 24 h of co-incubation with monocultures of different oral species (mean + SD; *$p < 0.05$). The black bars represent data from experiments performed without pre-incubation of the microbial cells on the insert, the white bars represent data from experiments with a 4 h pre-incubation step of the microbiota before confrontation with the epithelial cells.

Supplementary tables

Table S1a: p-Values of the ANOVA-analysis with Bonferroni correction of the wound healing assays, comparing data generated for the control cells (TR146 cells exposed to an insert without microbiota) with data obtained for the TR146 cells that were co-cultured with different oral microbial species. **b:** Microbial counts of the different oral species present on the insert at time 0 and after 24 h of co-culture with TR146 epithelial cells (mean ± SD; N.D. = not detected). Data shown from experiments performed with and without a 4 h pre-incubation step of the microbial inserts prior to confrontation with TR146 cells.

a

**P-values wound healing after 24 h**	**Pre-incubation 0 h**	**4 h**
S. salivarius	0.587	< 0.001
S. oralis	0.022	0.029
S. mitis	0.054	0.081
S. pyogenes	1.000	0.200
L. salivarius	0.070	< 0.001
L. oralis	0.933	0.206
L. plantarum	0.003	0.206
N. mucosa	0.517	0.580
K. oxytoca	0.007	0.001

b

**Bacterial counts after 24 h (log CFU/filter)**	**Pre-incubation 0 h**	**4 h**
S. salivarius	1.63 ± 0.92	6.27 ± 0.41
S. oralis	1.79 ± 0.05	3.45 ± 0.03
S. mitis	1.69 ± 0.30	3.08 ± 0.42
S. pyogenes	N.D.	3.80 ± 0.62
L. salivarius	7.34 ± 0.06	7.67 ± 0.12
L. oralis	5.74 ± 0.05	5.69 ± 0.08
L. plantarum	7.67 ± 0.08	7.65 ± 0.11
N. mucosa	4.13 ± 0.46	5.09 ± 0.55
K. oxytoca	8.66 ± 0.14	8.72 ± 0.13

Table S2: Concentrations of IL-1β, IL-6, TNF-α and Rantes present in the co-culture medium of TR146 cells exposed to different oral microbial species (with a 4 h pre-incubation step for the microbiota). p-Values of the ANOVA analysis with Bonferroni correction for comparison of the data generated for control cells (TR146 cells exposed to an insert without microbiota) and the data obtained from TR146 cells that were co-cultured with different oral microbial species.

	TNF-α (pg/mL)		IL-1ß (pg/mL)		IL-6 (pg/mL)		Rantes (pg/mL)	
	mean ± SD	p-value	mean ± SD	p-value	mean ± SD	p-value	mean ± SD	p-value
Control	0.390 ± 0.184		4.450 ± 0.820		179.380 ± 22.882		61.720 ± 5.572	
K. oxytoca	0.435 ± 0.120	1.000	5.390 ± 0.240	1.000	133.745 ± 21.786	0.726	32.950 ± 6.590	0.070
N. mucosa	0.717 ± 0.046	0.065	5.853 ± 1.515	1.000	243.530 ± 30.689	0.192	65.370 ± 5.284	1,000
S. salivarius	0.460 ± 0.052	1.000	3.497 ± 0.823	1.000	114.287 ± 21.783	0.182	45.523 ± 11.336	0.416
Control	0.573 ± 0.215		5.570 ± 1.889		144.247 ± 35.721		83.333 ± 17.594	
S. mitis	0.530 ± 0.105	1.000	7.927 ± 1.033	0.187	88.623 ± 12.271	0.093	44.363 ± 9.016	0.020
S. pyogenes	0.257 ± 0.150	0.165	2.363 ± 0.387	0.063	179.157 ± 18.484	0.386	42.807 ± 4.516	0.016
Control	0.200 ± 0.089		4.800 ± 1.457		83.793 ± 9.326		59.413 ± 3.804	
L. oralis	0.347 ± 0.085	0.428	3.820 ± 0.339	1.000	78.247 ± 3.974	1.000	56.193 ± 3.563	1.000
L. pyogenes	0.200 ± 0.137	1.000	4,503 ± 0.811	1.000	87.053 ± 15.780	1.000	60.237 ± 7.383	1.000
Control	0.300 ± 0.220		6.200 ± 1.429		150.237 ± 33.858		69.740 ± 6.914	
S. oralis	0.317 ± 0.067	0.906	10.480 ± 0.469	0.008	126.847 ± 16.603	0.343	53.070 ± 2.363	0.017
Control	0.157 ± 0.023		1.843 ± 0.257		92.993 ± 19.647		48.657 ± 1.000	
L. salivarius	0.215 ± 0.064	0.407	2.673 ± 0.682	0.120	80.493 ± 10.583	0.387	32.373 ± 8.381	0.076

Table S3: Results of the MALDI-TOF-analysis of the conditioned medium fraction < 3 kD of control cells (TR146 cells exposed to an insert without microbiota) and *K. oxytoca*-exposed cells. Presence (large peak: +; small peak: (+); absence (-)) of different peptides is shown together with the fraction in which these were found. One peptide was more abundant in the eluate of the *K. oxytoca*-conditioned medium (*).

Peptide	*Control*	Fraction	*K. Oxytoca*	Fraction
970.4	+	Drain	+	Drain
993	+	Washing step 50% MeOH	+	Washing step 50% MeOH<br>Eluate
996.5	+	Eluate	(+)	Drain
1051	(+)	Eluate	-	
1158	+	Washing step 50% MeOH	+	Eluate
1165.6	+	Eluate	-	
1186.6	+	Drain	+	Drain
1208.5	+	Washing step 10% MeOH	+	Washing step 10% MeOH
1236.6	+	Washing step 10% MeOH<br>Washing step 50% MeOH	+	Washing step 10% MeOH<br>Eluate
1257.5	+	Washing step 50% MeOH	+	Washing step 50% MeOH<br>Eluate
1279.6	(+)	Eluate	-	
1534.6	+	Washing step 50% MeOH	+	Eluate
* 1605.6	(+)	Washing step 50% MeOH<br>Eluate	+	Eluate
1732	+	Washing step 50% MeOH	+	Washing step 50% MeOH
